# Autophosphorylation of CaMKK2 generates autonomous activity that is disrupted by a T85S mutation linked to anxiety and bipolar disorder

**DOI:** 10.1038/srep14436

**Published:** 2015-09-23

**Authors:** John W. Scott, Elizabeth Park, Ramona M. Rodriguiz, Jonathan S. Oakhill, Samah M. A. Issa, Matthew T. O’Brien, Toby A. Dite, Christopher G. Langendorf, William C. Wetsel, Anthony R. Means, Bruce E. Kemp

**Affiliations:** 1 St Vincent’s Institute and Department of Medicine, University of Melbourne, 41 Victoria Parade, Fitzroy, 3065, Australia; 2 Department of Psychiatry and Behavioral Sciences, Mouse Behavioral and Neuroendocrine Core Facility, Duke University Medical Center, Durham, NC 27710; 3 Departments of Cell Biology and Neurobiology, Duke University Medical Center, Durham, NC 27710; 4 Department of Pharmacology and Cancer Biology, Duke University Medical Center, Durham, NC 27710.

## Abstract

Mutations that reduce expression or give rise to a Thr85Ser (T85S) mutation of Ca^2+^-CaM-dependent protein kinase kinase-2 (CaMKK2) have been implicated in behavioural disorders such as anxiety, bipolar and schizophrenia in humans. Here we report that Thr85 is an autophosphorylation site that endows CaMKK2 with a molecular memory that enables sustained autonomous activation following an initial, transient Ca^2+^ signal. Conversely, autophosphorylation of Ser85 in the T85S mutant fails to generate autonomous activity but instead causes a partial loss of CaMKK2 activity. The loss of autonomous activity in the mutant can be rescued by blocking glycogen synthase kinase-3 (GSK3) phosphorylation of CaMKK2 with the anti-mania drug lithium. Furthermore, CaMKK2 null mice representing a loss of function model the human behavioural phenotypes, displaying anxiety and manic-like behavioural disturbances. Our data provide a novel insight into CaMKK2 regulation and its perturbation by a mutation associated with behavioural disorders.

Anxiety and bipolar disorders are among the most prevalent mental health conditions in the world. Genome wide studies have identified the region of chromosome 12q24.31 as a susceptibility locus containing the Ca^2+^-calmodulin dependent protein kinase kinase-2 (*CaMKK2*) gene, which has been implicated in the pathogenesis of anxiety, bipolar disorder, and schizophrenia^1^,^2^,^3^. The intronic SNP rs1063843 is associated with reduced expression of CaMKK2 in schizophrenic patients^2^. A second CaMKK2 exonic SNP (rs3817190, minor allele frequency 0.38^1^,^4^) results in a T85S mutation linked to anxiety and bipolar disorder^1^,^3^,^4^.

CaMKK2 is a member of the Ca^2+^-calmodulin dependent protein kinase (CaMK) family and was originally identified as a CaMKI and CaMKIV upstream activating kinase^5^,^6^,^7^,^8^. It is also an upstream kinase for the metabolic stress-sensing enzyme AMP-activated protein kinase (AMPK) and the histone deacetylase Sirtuin 1 (Sirt1)^9^,^10^,^11^,^12^. Like other CaMK family members, CaMKK2 consists of unique N- and C-terminal sequences buttressing a mid-molecule catalytic domain and a regulatory module comprising overlapping autoinhibitory and calmodulin (CaM) binding sequences^13^. The autoinhibitory sequence is thought to occlude the active site in the absence of Ca^2+^-CaM; i.e. by an intrasteric autoinhibition mechanism that blocks access of substrates^14^. The Ca^2+^-CaM sensitivity of CaMKK2 is uniquely dependent on phosphorylation of Ser129, Ser133 and Ser137 located within a regulatory sequence N-terminal to the catalytic domain^15^. Phosphorylation of Ser137 by proline-directed kinases (Pro-K) primes CaMKK2 for subsequent phosphorylation on Ser133 and Ser129 by glycogen synthase kinase-3 (GSK3). Without phosphorylation at these sites, CaMKK2 exhibits increased autonomous activity in the absence of Ca^2+^-CaM^15^,^16^.

CaMKK2 is highly expressed in the nervous system and plays key roles in maintaining neuronal plasticity, as well as regulating axonal growth and dendrite formation^15^,^17^. One report has claimed genetic deletion of CaMKK2 impairs long-term memory formation in the hippocampus resulting from selective loss of long-term potentiation at hippocampal CA1 synpases^18^. In the hypothalamus, CaMKK2 is activated in response to the orexigenic hormone ghrelin, leading to activation of AMPK and appetite stimulation^19^. Prompted by the human genetic association studies we have now determined the functional consequences of the T85S mutation on human CaMKK2 activity and investigated the effect of CaMKK2 deletion on behavior in mice.

## Results and Discussion

**Figure 1:**
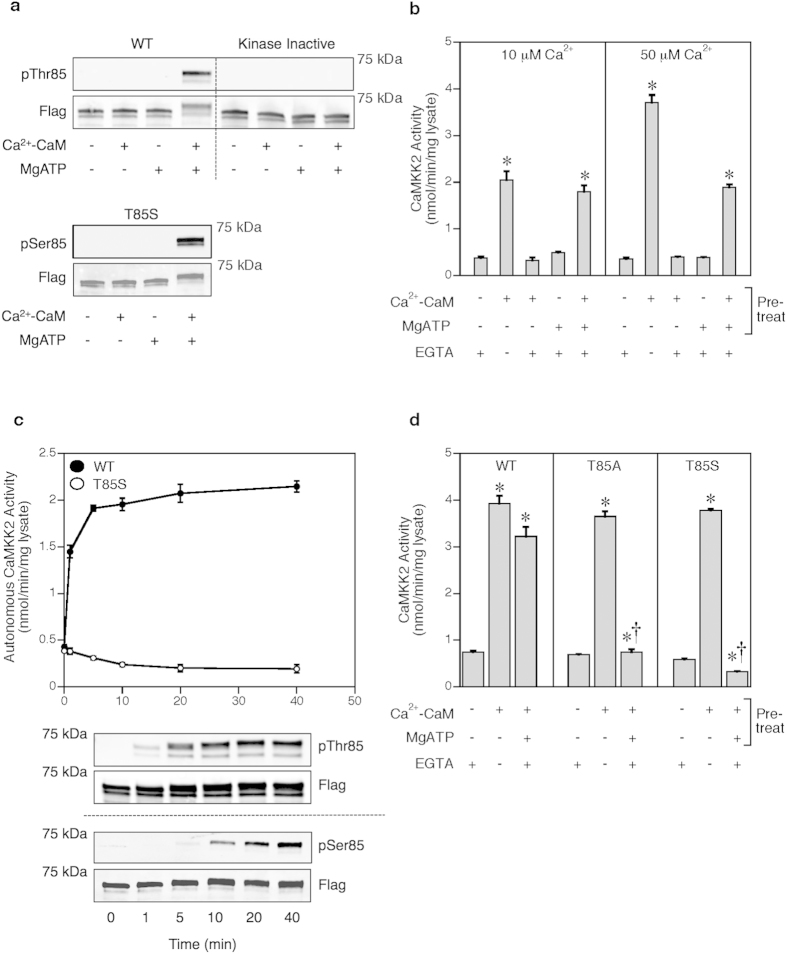
Autophosphorylation sustains CaMKK2 activation after Ca^2+^ withdrawal. (**a**) Autophosphorylation of wild-type CaMKK2 and T85S mutant in the presence or absence of Ca^2+^-CaM and MgATP for 40 min as shown using phospho-specific pThr85 and pSer85 antibodies on a representative cropped immunoblot. Total CaMKK2 was detected using anti-Flag antibody. (**b**) Effect of autophosphorylation on wild-type human CaMKK2 activity. The autophosphorylation reaction was performed in the presence or absence of 10 or 50 μM Ca^2+^, 1 μM calmodulin and 200 μM MgATP for 40 min, after which CaMKK2 activity was measured in the presence or absence of 1 mM EGTA. (**c**) Time-course of autophosphorylation of wild-type CaMKK2 or T85S mutant in the presence of 50 μM Ca^2+^-CaM and 200 μM MgATP. An aliquot was removed at the time points indicated and assayed for CaMKK2 activity in the presence of 1 mM EGTA, or immunoblotted as described above. (**d**) Effect of T85S and T85A mutations on CaMKK2 activity and Ca^2+^-CaM dependence before and after autophosphorylation. CaMKK2 activity was measured in the presence or absence of 1 mM EGTA. Data are presented as mean ± SEM; n = 4 for each figure. Statistical analysis was performed by one-way ANOVA. *p < 0.001, vs the control within the same group; †p < 0.001, vs the WT control within the same condition.

Using purified enzyme and pThr85 or pSer85 phosphospecific antibodies, we found both Thr85 in human WT CaMKK2 and Ser85 in the T85S mutant underwent autophosphorylation in the presence of Ca^2+^-CaM and MgATP, but not when incubated with MgATP alone or in a kinase inactive mutant (D312A) of CaMKK2 (Fig. 1a; Supplementary Fig. 1a,b; Supplementary Fig. 2). Autophosphorylation plays an important role in the function of other members of the CaMK protein kinase subfamily. For example Ca^2+^-CaM stimulates CaMKII autophosphorylation of Thr287, which sustains kinase activation after cessation of the Ca^2+^ signal^20^. We therefore investigated whether autophosphorylation of Thr85 similarly permits increased autonomous activity of CaMKK2. Autophosphorylation of CaMKK2 in the absence of Ca^2+^-CaM caused a modest increase (1.6 fold) in kinase activity; however, autophosphorylation in the presence of Ca^2+^-CaM yielded a much larger activation (~5-fold) that persisted even in the presence of the Ca^2+^-chelator EGTA (Fig. 1b). By comparison, EGTA blocked activation of CaMKK2 by Ca^2+^-CaM in the absence of autophosphorylation. The level of autonomous activity generated by autophosphorylation is similar to the maximal activation achieved with 10 μM Ca^2+^, but ~50% less than with 50 μM Ca^2+^. Since the concentration range of Ca^2+^ in neurons is between ~0.1–10 μM^21^, the amplitude of autonomous activation (~5-fold activation) is similar to the Ca^2+^-dependent activity achievable over the cellular Ca^2+^ signaling range (Supplementary Fig. 3). Autophosphorylation generates autonomous activity in a time-dependent manner (Fig. 1c, upper panel), and this is matched by Thr85 autophosphorylation in WT CaMKK2, and Ser85 autophosphorylation in the T85S mutant (Fig. 1c, lower panel). Dilution of WT CaMKK2 had no effect on the degree of autonomous activity achieved with autophosphorylation, consistent with an intramolecular (*cis*) autophosphorylation mechanism (Supplementary Fig. 4). Autonomous activity in the presence of EGTA was lost in a CaMKK2 T85A mutant despite it being normally activated by Ca^2+^-CaM (Fig. 1d). The T85S mutant was also normally activated by Ca^2+^-CaM; however, chelation of Ca^2+^ after autophosphorylation gave rise to significant (43%) inhibition of CaMKK2 activity (Fig. 1d). These data indicate that autophosphorylation of Thr85 endows CaMKK2 with a molecular memory that enables sustained autonomous activation following an initial, transient Ca^2+^ signal. Conversely, autophosphorylation of Ser85 fails to maintain similar molecular memory of CaMKK2. Other examples of correspondingly high levels of functional discrimination between phosphorylated Thr and Ser residues have been reported, including the insulin receptor kinase, forkhead-associated (FHA) domain recognition of pThr, and for the protein phosphatase-1 regulatory subunit DARPP-32^22^,^23^,^24^.

**Figure 2:**
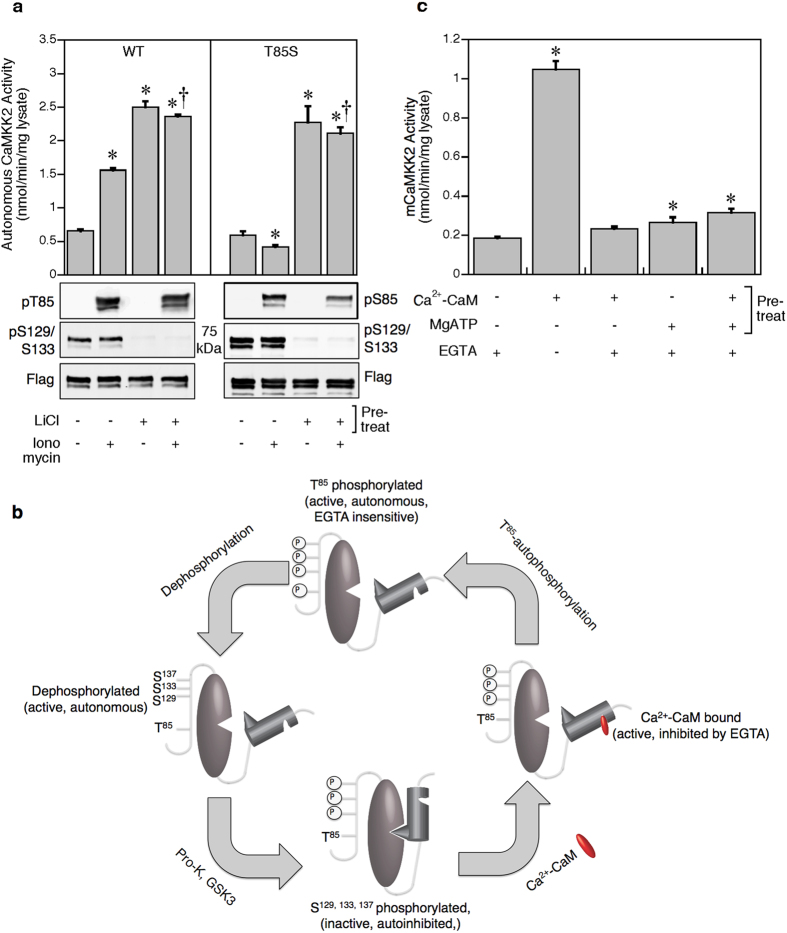
Lithium treatment rescues the inhibitory effect of Ser85 autophosphorylation of the T85S mutant. (**a**) Autonomous activity of CaMKK2 from cells treated with 10 mM lithium chloride and 10 μM ionomycin. Kinase activity was measured in the presence of 1 mM EGTA. A representative cropped immunoblot shows the phosphorylation status of Thr85, Ser85 and Ser129/Ser133 in response to the treatments. (**b**) Schematic illustrating regulation of CaMKK2 by Ca^2+^-CaM and reversible phosphorylation/autophosphorylation. (**c**) Effect of autophosphorylation on wild-type mouse CaMKK2 activity. Data are presented as mean ± SEM; n = 4 for each figure. Statistical analysis was performed by one-way ANOVA. *p < 0.001, vs the control within the same group; †p < 0.001, vs ionomycin in the absence of lithium within the same group.

Lithium is used clinically to treat bipolar disorder and is a GSK3 inhibitor^25^. Previously we found that CaMKK2 is constitutively phosphorylated on Ser129 and Ser133 by GSK3, which suppresses autonomous activity and sensitizes the enzyme to Ca^2+^-CaM^15^. Wild-type CaMKK2 from transfected COS7 cells treated with ionomycin displayed increased autonomous activity, whereas the T85S mutant had decreased activity (Fig. 2a). Lithium chloride treatment similarly increased autonomous activity of WT CaMKK2, but also reversed the inhibitory effect of ionomycin on the T85S mutant to generate autonomous activity that approximated WT levels. This indicates that Ser129 and Ser133 dephosphorylation is functionally dominant as lithium treatment increased autonomous activity in the T85S mutant despite the fact that Ser85 is autophosphorylated. Altogether, our data show that human CaMKK2 has two distinct mechanisms to achieve autonomous activity i.e Thr85 autophosphorylation or Ser129/Ser133 dephosphorylation (Fig. 2b). Multiple mechanisms to achieve autonomous activity are also seen with CaMKII that include methionine oxidation of Met281/Met282, O-GlcNAcylation of Ser279, and binding of the T-site of CaMKII to the NR2B subunit of the NMDA receptor^26^,^27^,^28^.

Alignment of the human CaMKK2 amino acid sequence with other mammalian species revealed that Thr85 is restricted to primates (Supplementary Fig. 5). However, there is no absolute requirement for interspecies conservation of functional phosphorylation sites^29^, particularly those located within poorly conserved sequences such as Thr85 in CaMKK2. The gain or loss of phosphorylation sites in rapidly evolving unstructured regions is postulated to facilitate the evolution of new signaling pathways and control mechanisms^30^. Consistent with the absence of an equivalent threonine, we found that autophosphorylated mouse CaMKK2 had very little autonomous activity after Ca^2+^ chelation with EGTA (Fig. 2c). However, mouse CaMKK2 can still achieve autonomous activity via dephosphorylation of Ser129/Ser133 in response to lithium (Supplementary Fig. 6).

**Figure 3:**
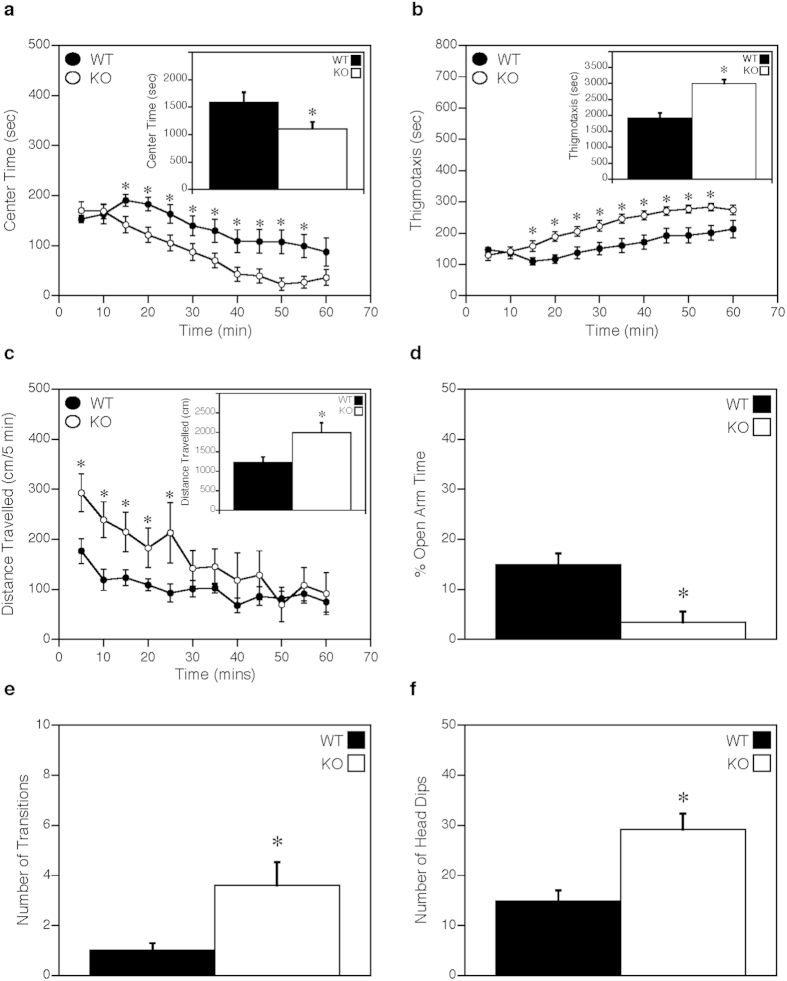
CaMKK2 KO mice exhibit anxiety-like behaviour. (**a**) Time (min) spent in the center of the open field measured over 1 hr. Inset is the cumulative time spent in the center zone over the 1 hr test. (**b**) Time (min) spent along the perimeter (thigmotaxis) of the open field measured over 1 hr. Inset is the cumulative time spent along the open field perimeter over the 1 hr test. (**c**) Locomotor activity expressed as distance travelled (cm) in the open field measured over 1 hr. Inset is the cumulative distance travelled over the 1 hr test. (**d**) Percentage of total time spent in the open areas of the elevated zero maze. (**e**) Number of transitions between the open and closed quadrants of the elevated zero maze. (**f**) Number of head-dips over the edge of the open areas of the zero maze. Data are presented as mean ± SEM; n = 10 for WT and CaMKK2 KO. Statistical analysis performed by repeated measures ANOVA (panels **a**–**c**) or t-tests (Insets for panels **a**–**c**; panels **d**–**f**). *p < 0.05, WT vs KO.

As Thr85 is not conserved in rodents we are unable to generate T85S knock-in mice to directly study the effects of this mutation on behaviour. Nevertheless, numerous studies indicate a strong association between reductions in CaMKK2 expression and behavioural disorders^1^,^2^,^3^; accordingly, we used CaMKK2 null mice as a constitutive loss of function model to study behaviour. Anxiety-like behaviors of the CaMKK2 null (KO) mice were evaluated using the open field and elevated zero maze tests. In the open field, the KO mice display anxiety-like behaviours relative to the wild-type (WT) controls as evidenced by reduced time spent in the centre zone of the open field with an increase time spent at the perimeter (thigmotaxis) (Fig. 3a,b). The KO mice also show an overall enhancement in locomotor activity although they engage in similar rearing and stereotypical activities as the WT controls (Fig. 3c; Supplementary Fig. 7a,b). In the elevated zero maze, the KO mice spend less time in the open areas, make more transitions between the two closed quadrants by traversing an open area, and display significantly more head-dips than WT mice (Fig. 3d–f). However, in the maze there were no genotype differences in the incidences of freezing behaviors or the frequencies of stretch-attend postures (data not shown), which may be a reflection of specific brain regions relying more on CaMKK2 signalling than others. Overall, these data show that the KO mice are hyperactive, exhibit anxiety-like phenotypes, and engage in more risk assessing behaviors.

**Figure 4:**
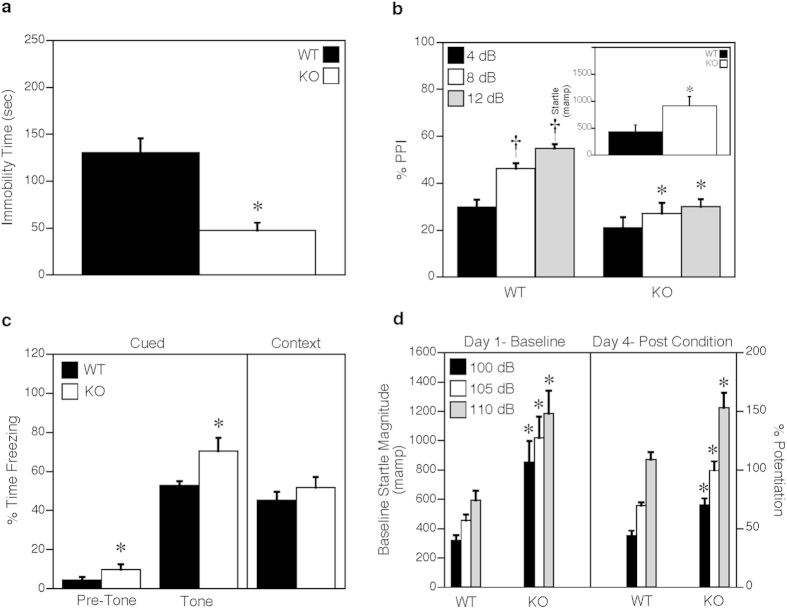
CaMKK2 KO mice display reduced immobility in tail suspension and abnormal fear responses. (**a**) Time (sec) spent immobile during the tail suspension test. (**b**) Percent prepulse inhibition of the acoustic startle response. Insert shows the baseline startle response to a 120 dB acoustic stimulus. (**c**) Freezing behavior in cued and contextual fear conditioning 24 hr after a single CS-US pairing. (**d**) Baseline startle responses to three intensities of acoustic stimuli on day 1, and the percent fear-potentiated startle response at testing on day 4. Data are presented as mean ± SEM; n = 10 for WT and CaMKK2 KO. Statistical analyses of WT vs KO was performed by t-tests in (panel **a** and panel **c**) (contextual fear conditioning) and by repeated measures ANOVA for the other panels. *p < 0.05, WT vs KO; †p < 0.05, within genotype effect for PPI, 4 vs 8 and 12 dB.

Immobility during the tail suspension test is used as an indicator of depression, whereas persistent struggling can indicate hyperactivity or manic-like responses in mice^31^. Consistent with the hyperactivity in the open field, immobility in the CaMKK2 KO mice was significantly reduced relative to their WT littermates (Fig. 4a). In the prepulse inhibition (PPI) test, startle responses to the 120 dB acoustic stimulus were significantly augmented in the CaMKK2 KO mice (Fig. 4b, inset) and these mutants also showed reduced PPI (Fig. 4b), which is observed in humans during schizophrenic and manic episodes^32^.

Since rodents that are anxious also display abnormal fear responses^33^, we evaluated fear conditioning of the CaMKK2 KO mice. Here, freezing behaviors were increased in the KO mice during testing for cued conditioning at the pre-cue as well as the cue intervals (Fig. 4c, left). By comparison, the genotypes were not differentiated in their responses to context testing (Fig. 4c, right). The distinctions between cued and context testing suggest that amygdala function may be abnormal. To examine this possibility, mice were evaluated in fear-potentiated startle. In this test, the CaMKK2 KO mice displayed enhanced basal startle responses to the acoustic stimuli on the first day of testing (Fig. 4d, left) as well as during preconditioning potentiation on day 2 (Supplementary Fig. 8). Following conditioning on day 4, fear-potentiated startle responses were augmented in the KO compared to the WT controls (Fig. 4d, right). Collectively, these findings indicate that the amygdala is hyper-responsive in the CaMKK2 KO mice and they have abnormal fear memories.

Of the multiple genes that have been identified from genome wide studies to be associated with the pathogenesis of anxiety, bipolar disorder, and schizophrenia, CaMKK2 and its interactome are prominent^2^. Multiple convergent lines of evidence point strongly to reductions in CaMKK2 expression/activity with behavioural disorders: 1) Our results show that CaMKK2 null mice exhibit behavioural disturbances similar to those observed in humans with behavioural disorders; 2) We also show the T85S mutant linked with bipolar and anxiety in humans is unable to generate autonomous activity via Thr85 autophosphorylation and causes a intermittent loss of activity; 3) Reduced expression of CaMKK2 is associated with schizophrenia in seven diverse human populations^2^; 4) Mice treated with methamphetamine to induce bipolar-like symptoms have decreased CaMKK2 expression, but normal expression is maintained with co-treatment of the mood stabiliser valproate^3^. Our data provide a mechanistic insight into the regulation of CaMKK2 activity and its perturbation by a mutation associated with behavioural disorders.

## Methods

### Animals

CaMKK2 null (KO) mice were generated via homologous recombination as described previously and backcrossed nine generations onto C57Bl/6^19^. The KO mice and wild-type controls (8–12 wks old) were segregated by genotype and housed 4–5 animals per cage in a humidity- (45%) and temperature-controlled (22 °C) room. A 14:10 hr light:dark cycle (lights on at 0700 hr) was used, and all behavioral testing was conducted between 0900 and 1700 hr. Mice were maintained on standard rodent chow (Richmond Diet 5001, Lab Diet Inc., Richmond, VA) and tap water *ad libitum*. All experiments were conducted using young adult male WT and KO littermates in accordance with National Institutes of Health guidelines for the care and use of laboratory animals and with an approved protocol from the Duke University Institutional Animal Care and Use Committee.

### Open field test

Spontaneous activity was monitored in the open field (21 × 21 × 30 cm) apparatus (AccuScan Instruments, Columbus, OH) at 5 min intervals over a 1 hr period as described^34^,^35^. Mice were acclimated to the room at least 2 hr before the experiment and testing was under 340-lux illumination. Using the VersaMax program (AccuScan Instruments), spontaneous locomotor activity was measured as the total distance traveled in cm and rearing as the total number of vertical beam-breaks. Stereotypical activity was evaluated by repetitive beam-breaks within a limited location with intervals <1 sec. Anxiety-like behavior was assessed as the percentage of time spent in the center zone compared to the periphery of the open field.

### Elevated zero maze test

Mice were evaluated for anxiety-like behaviors using the elevated zero-maze as described^34^,^35^. Mice were placed into a closed quadrant of the zero maze and activity was videotaped for 5-min under 50–60 lux illumination. Trained observers, blinded to the animal genotypes, scored the videos using the Observer program (Noldus Information Technologies, Leesburg, VA). The scored activities included percent time spent in the open areas, total number of transitions through open areas between the two closed quadrants, the frequencies of head-dipping and stretch-attend postures, and time spent freezing. Anxiety was operationally defined as reduced time spent in the open areas.

### Tail suspension test

Mice were tested in a tail suspension apparatus (Med Associates, St. Albans, VT) over 6 min as described^34^. To control for the magnitude of struggling activity by the Tail Suspension Software the body weight of each mouse was entered to control for the magnitude of struggling activity by the Tail Suspension Software. Mice were suspended from their tails by tape and the duration of immobility was used as an index of depressive-like activity and was defined as the absence of any initiated movements and included passive swaying.

### Emotional learning and memory

Mice were examined in conditioned fear across three days as previously described^36^,^37^. On day 1, mice were placed into an automated fear-conditioning chamber (Med Associates) and after 2 min a 72 dB, 2900 HZ tone (CS) was presented for 30 sec that terminated with a 2-sec 0.4 mA scrambled foot-shock (US). Mice remained in the chamber for additional 30 sec before they were returned to their home cages. Twenty-four hr following conditioning, the mice were examined for contextual fear. The animals were returned to the same chamber in which they were conditioned, in the absence of the CS and US for 5-min. On the third day, mice were tested for cued fear conditioning. Mice were placed into a novel chamber and after 2 min, then the CS was presented for 3 min. No US was given. Time spent in freezing was scored by trained observers blinded to the genotype of the animal using Observer software (Noldus Information Technologies) and expressed as the percent time freezing relative to the duration of the interval (pre-CS or CS for cued testing) or the test (context). Freezing was defined as the absence of movement, aside from that required for respiration.

### Fear-potentiated startle

Fear potentiated startle (FPS) was conducted over 4 days in Med Associates chambers^37^. Mice were acclimated to the chamber for 5 min each day before conditioning or testing. On day 1, baseline startle responses were measured by presenting 40 msec acoustic white-noise stimuli of different intensities (100, 105, and 110 dB). On day 2, one-half of the startle stimuli were paired with a 30 sec, 12-kHz, 70–dB tone (CS) to assess naturally occurring potentiation of the startle response by the CS; the other half received the startle stimuli without the CS. On day 3, mice were conditioned with 10 pairings of the CS with a 0.25-sec 0.4-mA scrambled foot-shock (UCS). On day 4, the mice were examined for fear potentiation of their startle responses under the same procedure as that for day 2. The magnitude of response to the CS was calculated as: [(average tone + startle stimulus responses–average startle-only responses)/average startle-only responses] × 100.

### Expression and purification of recombinant CaMKK2

COS7 cells were grown in DMEM (Sigma) media with 10% fetal calf serum at 37 °C with 5% CO_2_. Cells were transfected at 60% confluency with 1 μg of pcDNA3 containing C-terminal Flag-tagged human or mouse CaMKK2 or various point mutants using FuGene 6 (Roche). Transfected cells were harvested after 48 hr by washing with ice-cold phosphate-buffered saline (PBS) followed by rapid lysis *in situ* using 1 ml of lysis buffer (50 mM Tris HCl [pH 7.4], 150 mM NaCl, 50 mM NaF, 1 mM NaPPi, 1 mM EDTA, 1 mM EGTA, 1 mM DTT, 1% [v/v] Triton X-100) containing protease inhibitors (Roche).

### Cell treatments with lithium chloride and ionomycin

Transfected COS7 cells (48 hr post-transfection) were pre-treated with LiCl (10 mM) for 1 hr, after which they were incubated for a further 30 min with 10 μM ionomycin (Sigma) and then harvested as described above. Cellular debris was removed by centrifugation and total protein was determined using the Bradford protein assay (Pierce). Recombinant CaMKK2 was purified from 1.5 mg of cell lysate using 10 μl of anti-Flag agarose (50% v/v) (Sigma) pre-equilibrated in lysis buffer, followed by successive washes in lysis buffer containing 1 M NaCl, and finally into 50 mM HEPES [pH 7.4]. The beads were then sedimented by centrifugation and used in a kinase assay or for immunoblotting.

### CaMKK2 activity assay

CaMKK2 activity was measured by its ability to phosphorylate a synthetic peptide substrate (LSNLYHQGKFLQTFCGAPLYRRR) corresponding to the activation loop residues 196–215 of human NuaK2, except that serine-212 was substituted with an alanine to prevent phosphorylation of this residue by proline-directed kinases. The peptide also contained three additional arginine residues at the C-terminus to promote binding of the peptide to P81 phosphocellulose paper. For a standard 30 μl assay, 10 μl of recombinant CaMKK2 immobilised on anti-Flag agarose beads (50% v/v) was incubated in assay buffer (50 mM HEPES [pH 7.4], 1 mM DTT, 0.02% [v/v] Brij-35) containing 200 μM peptide substrate, 10 or 50 μM CaCl_2_, 1 μM calmodulin (Sigma), 200 μM [γ-32P]-ATP (Perkin Elmer) and 5 mM MgCl_2_. Reactions were incubated at 30 °C for 10 min, after which they were terminated by spotting 15 μl onto P81 phosphocellulose paper and washing extensively in 1% phosphoric acid. Radioactivity was quantified by scintillation counting. Activity was corrected for variations in CaMKK2 expression between samples by immunoblotting using an anti-Flag antibody. For autophosphorylation reactions, 50 μl of anti-Flag agarose immobilised CaMKK2 was incubated in assay buffer containing 200 μM ATP, 5 mM MgCl_2_, 10 or 50 μM CaCl_2_ and 1 μM calmodulin in a 25 μl reaction volume. Reactions were incubated at 30 °C for various times, after which the beads were washed successively in lysis buffer containing 1 M NaCl, and finally resuspended in 50 mM HEPES [pH 7.4] to achieve a 50% slurry. A 10 μl aliquot was removed and kinase activity measured in the presence of 1 mM EGTA. The autophosphorylation reactions were buffered with 25 μM EGTA, which was determined empirically to chelate trace amounts of contaminating Ca^2+^ in the assay components. This was essential to measure the effects on CaMKK2 activity of Ca^2+^ concentrations in the low micromolar physiological range^38^.

### Generation of phospho-specific antibodies

Phosphorylated peptides based on residues 80–91 surrounding either Thr85 in wild-type CaMKK2 (CEVPLDpTSGSQAR) or Ser85 in the T85S mutant (CEVPLDpSSGSQAR) were synthesized and coupled to keyhole limpet hemocyanin via the peptide N-terminal cysteine residue using the coupling reagent *N*-succinimidyl-3(-2-pyridyldithio)propionate. Rabbits were immunized with 2 mg of peptide conjugate initially in 50% (v/v) Freunds complete adjuvant and in 50% (v/v) Freunds incomplete adjuvant for subsequent immunizations. Rabbits were boosted fortnightly with 2 mg of peptide conjugate and bled 7 days after booster injections. The pThr85 and pSer85 antibodies were then purified from serum by peptide affinity chromatography.

### Western blotting

Purified CaMKK2 was denatured in SDS-PAGE sample buffer, separated by SDS-PAGE and transferred to Immobilon PVDF membranes (Millipore). Membranes were blocked for 1 hr in PBS/1% Tween-20 (PBS-T) supplemented with 5% non-fat milk. Primary antibodies were diluted in PBS-T containing 1% non-fat milk at the following dilution: mouse or rabbit anti-Flag (Cell Signaling; 100 ng/ml), rabbit anti-pThr85 (300 ng/ml), anti-pSer85 (3000 ng/ml) and mouse anti-pSer antibody (BD Bioscience; 250 ng/ml), which selectively detects phosphorylated Ser129/Ser133 on CaMKK2^15^. After incubation with primary antibody solutions for 1 hr, the membranes were briefly washed in PBS-T, and then incubated with appropriate fluorescently labeled secondary antibodies, either goat anti-mouse IgG IRDye800 or goat anti-rabbit IgG IRDye680. Membranes were then scanned with an Odyssey Infrared Imager (Li-Cor).

### Mass spectrometry

For phosphopeptide analysis, 1 μg of CaMKK2 was digested with trypsin and analyzed by reversed-phase nHPLC-ESI-MS/MS using an UltiMate 3000 Nano LC HPLC system (Dionex) directly connected to a Triple-TOF 5600 mass spectrometer (AB SCIEX) in direct injection mode. Peptide mixtures were resolved on an analytical nanocapillary HPLC column (100 μm i.d. × 15 cm) packed with C_18_ Acclaim PepMap100 (3 μm particle size, 100 Å pore size) using a 1–75% elution gradient of 98% acetonitrile/2% of 0.1% formic acid (v/v) in water at a flow rate of 250 nl/min. Mass spectrometric data were analyzed using the database search engine ProteinPilot and the Paragon algorithm.

### Statistical analyses

Data are presented as mean and SEM, and were analyzed using SPSS 11 (IBM SPSS Statistics, Chicago, IL). Independent measures t-tests were used when two groups were compared. Data with time-dependent repeated measures with the same animals were examined with repeated measures analyses of variance (RMANOVA), including open field, fear conditioning, and fear-potentiated startle tests. In these tests the within subject effects were either minutes (open field) or test session (fear conditioning, fear potentiated startle). The between subject effect was genotype. Statistical analysis of the biochemical experiments was performed by one- and two-way ANOVA. *Post-hoc* tests were by Bonferroni corrected pair-wise comparisons. In all cases, p < 0.05 was considered significant.

## Additional Information

**How to cite this article**: Scott, J. W. *et al.* Autophosphorylation of CaMKK2 generates autonomous activity that is disrupted by a T85S mutation linked to anxiety and bipolar disorder. *Sci. Rep.* **5**, 14436; doi: 10.1038/srep14436 (2015).

## Supplementary Material

Supplementary Information
