# Supplementary material for: Autophosphorylation of CaMKK2 generates autonomous activity that is disrupted by a T85S mutation linked to anxiety and bipolar disorder
Source: Sci Rep. 2015 Sep 23;5:14436. doi: 10.1038/srep14436 (PMC4585769; doi:10.1038/srep14436)
Supplement: Supplementary Information [file srep14436-s1.pdf]

SUPPLEMENTARY INFORMATION

Autophosphorylation of CaMKK2 generates autonomous activity that is disrupted by a T85S mutation linked to anxiety and bipolar disorder

John W. Scott, Elizabeth Park, Ramona M. Rodriguiz, Jonathan S. Oakhill, Samah M. A. Issa, Matthew T. O'Brien, Toby A. Dite, Christopher G. Langendorf, William C. Wetsel, Anthony R. Means and Bruce E. Kemp

a

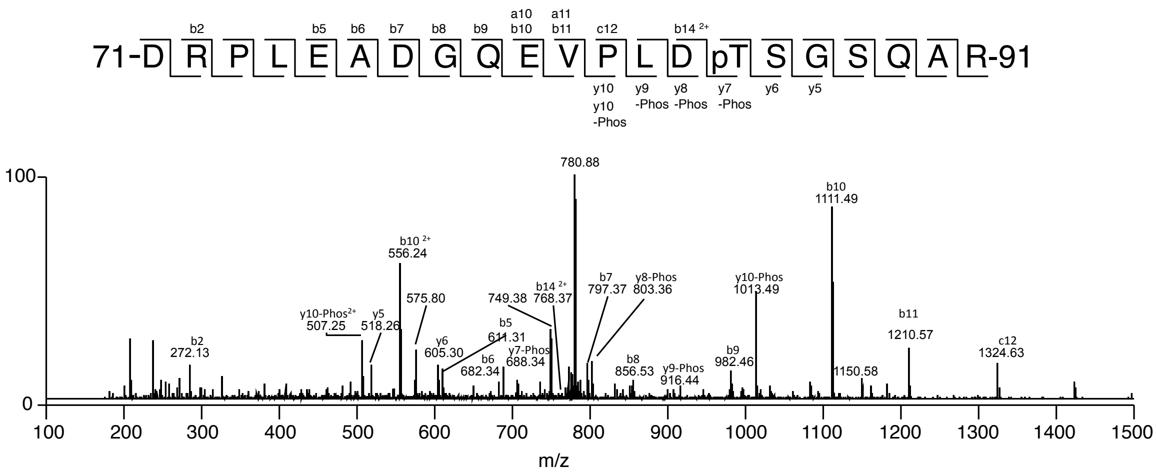

b

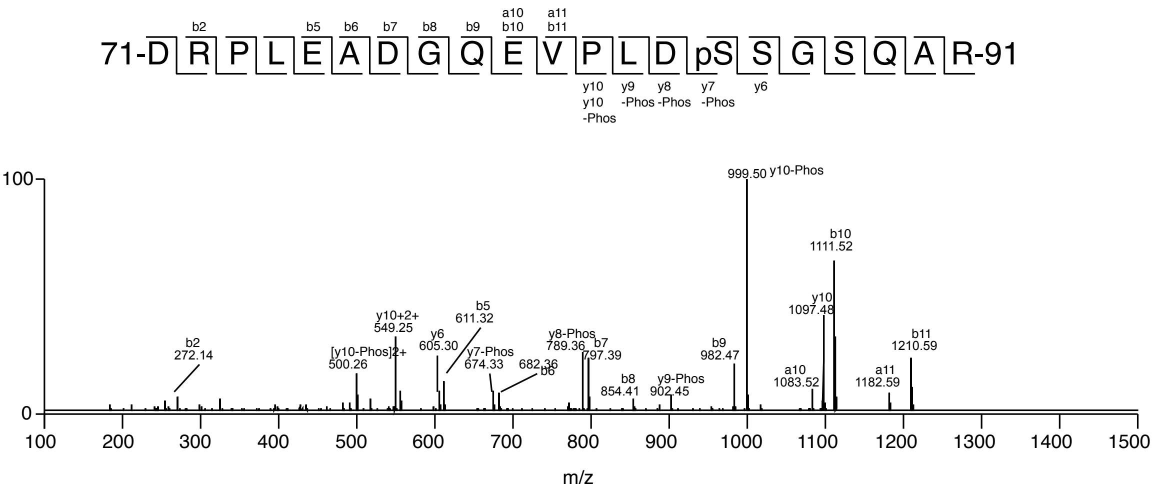

Supplementary Figure 1. Thr85 is a Ca^{2+}-CaM dependent autophosphorylation site. (**a**) Wild-type CaMKK2 that was autophosphorylated in the presence of MgATP and Ca^{2+}-CaM was subjected to trypsin digestion and LC-MS/MS mass spectrometry analysis. The masses of the b and y ion series provide direct evidence for phosphate incorporation onto Thr85. (**b**) The masses of the b and y ion series showing phosphate incorporation onto Ser85 in the T85S mutant.

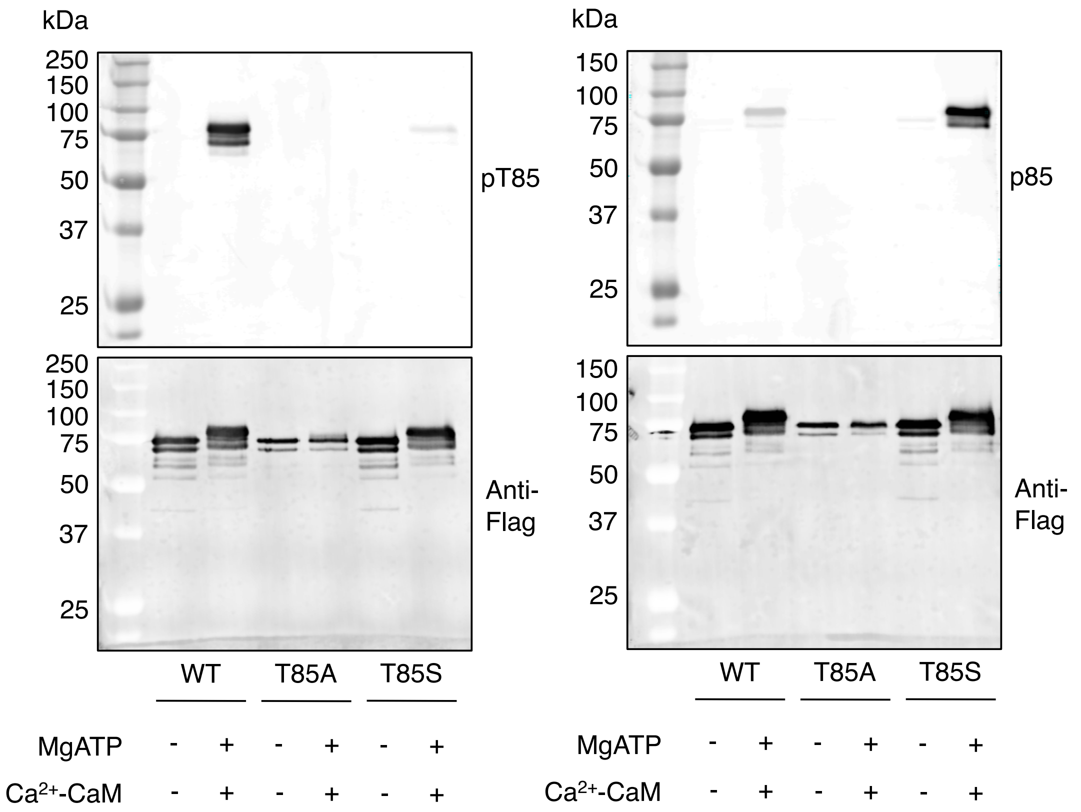

Supplementary Figure 2. Characterisation of phosphospecific pThr85 and pSer85 antibodies. CaMKK2 WT, T85A or T85S mutants were incubated in the presence or absence of MgATP and Ca^{2+}-CaM for 40 min. Representative immunoblots of the reactions were probed with either rabbit anti-pThr85 (300 ng/ml) or rabbit anti-pSer85 (3000ng/ml), and mouse anti-Flag (100 ng/ml), and visualised using goat anti-rabbit IgG IRDye680 or goat anti-mouse IgG IRDye800 secondary antibodies.

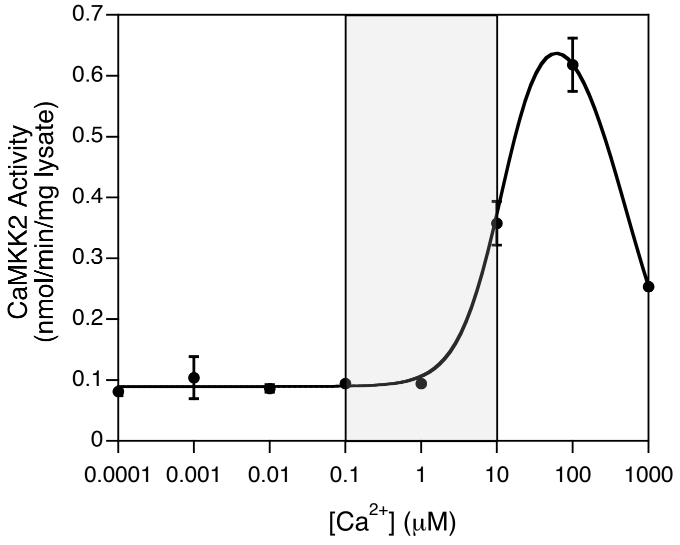

Supplementary Figure 3. Ca^{2+}-concentration dependence of CaMKK2. Activity of wild type CaMKK2 was measured over a range of Ca^{2+}-concentrations (0-1000 μM) in the presence of 1 μM calmodulin. The cellular Ca^{2+} (0.1-10 μM) concentration range is highlighted in grey. Data are presented as mean ± SEM; n=2.

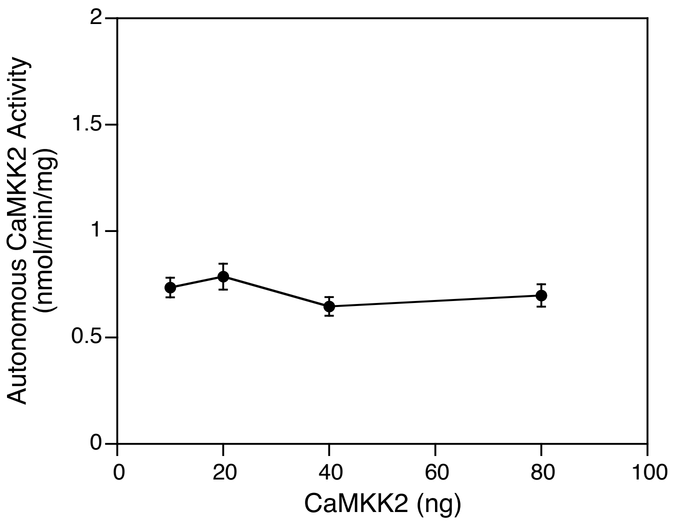

Supplementary Figure 4. Autophosphorylation of Thr85 occurs by an intramolecular *cis* mechanism. Wild-type CaMKK2 was serially diluted then subjected to autophosphorylation in the presence of MgATP and Ca^{2+}-CaM for 10 min. Kinase activity was measured in the presence of 1 mM EGTA and then corrected for dilution; the data are expressed as autonomous CaMKK2 activity. Data are presented as mean ± SEM; n=4 and statistical analysis was performed by one-way ANOVA.

```
     Human   PLEADGQEVPLDT—SGSQARPHLSGRK
    Baboon   PLEADGQEVPLDT—SGSQARPHLSGRK
Chimpanzee   PLEADGQEVPLDT—SGSQARPHLSGRK
   Gorilla   PLEADGQEVPLDT—SGSQARPHLSSRK

    Bovine   PLEAHGGEITLDA—SGSQARPHLSGRK
    Canine   PREAEGRKVPLDASASGSQARPQLCSR
       Rat   PLEADGQELPLDA—SEPESRSLLSGGK
     Mouse   PPEADGQELPLEA--SDPESRSPLSGR
```

Supplementary Figure 5. Sequence alignment of amino acids surrounding Thr85 from other mammalian species. Amino acid sequence alignment of residues 73-98 surrounding Thr85 of human CaMKK2 with orthologues from various mammalian species. The alignment was generated using Clustal Omega (http://www.ebi.ac.uk/Tools/msa/clustalo).

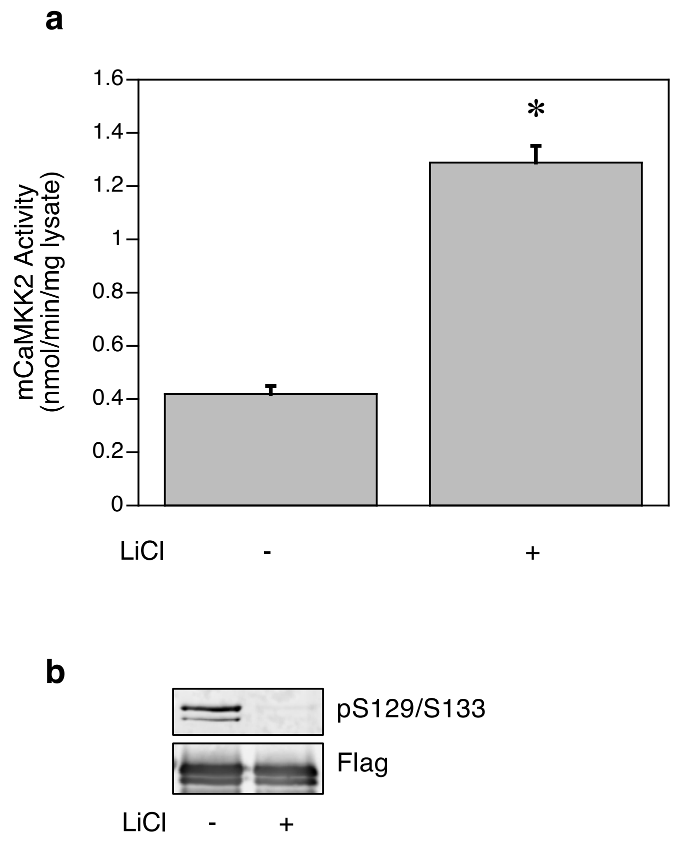

Supplementary Figure 6. Lithium treatment increases autonomous activity of mouse CaMKK2. (**a**) Autonomous activity of mouse CaMKK2 from cells treated with 10 mM lithium chloride for 1 hr. Kinase activity was measured in the presence of 1 mM EGTA. (**b**) A representative cropped immunoblot showing the phosphorylation status of Ser129/Ser133 in response to the lithium treatment. Data are presented as mean ± SEM; n=4 for each figure. Statistical analysis was performed by one-way ANOVA. *p<0.001, vs the control.

a

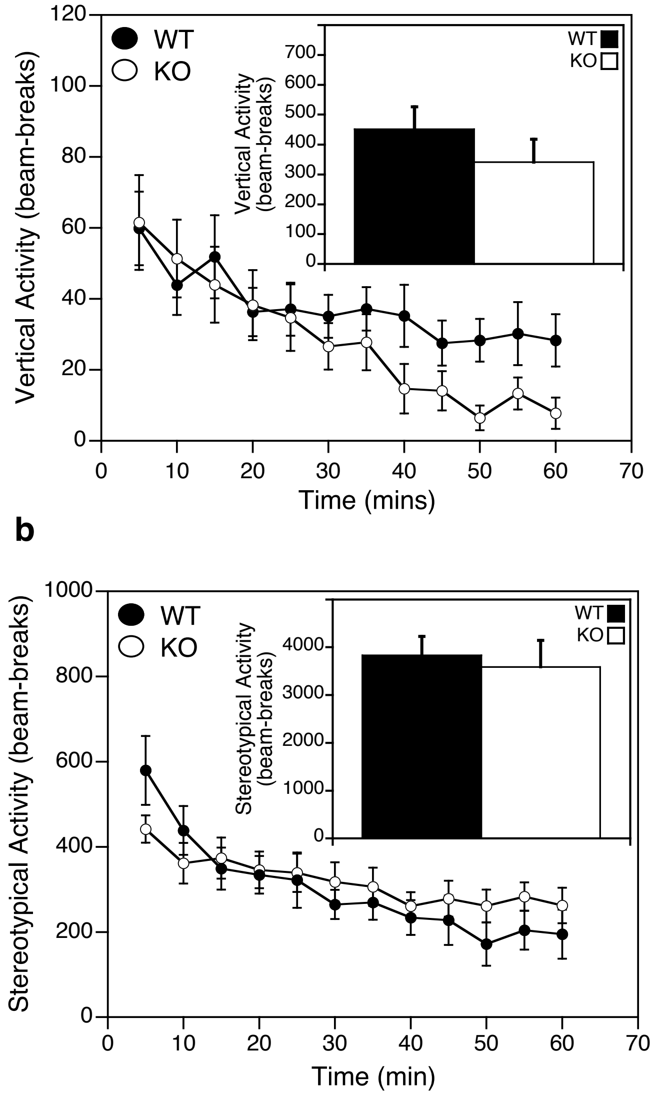

Supplementary Figure 7. CaMKK2 KO mice have normal rearing and stereotypical activities. (**a**) Rearing activity was measured as the number of vertical beam-breaks in 1 hr. (**b**) Stereotypical activity was measured as the number of repetitive beam-breaks <1 sec in 1 hr. Data are presented as mean ± SEM; n=10 for WT and CaMKK2 KO. Statistical analyses were performed by repeated measures ANOVA or t-tests (insets).

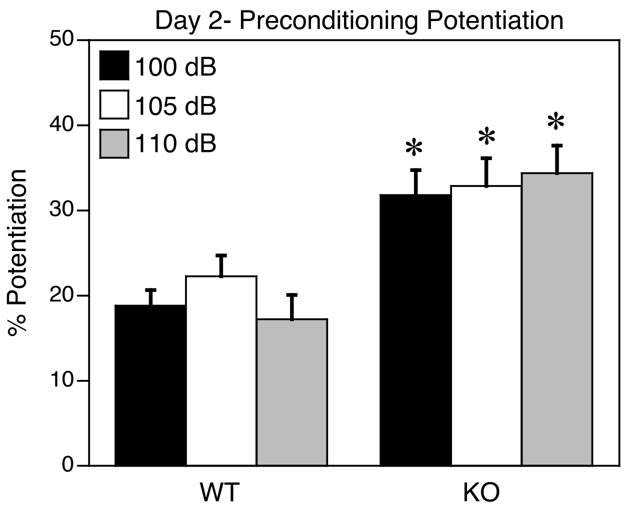

Supporting Figure 8. CaMKK2 KO mice have normal responses to day 2 preconditioning potentiation. On day 2 of fear-potentiated startle training, there are no genotype differences. Data are presented as mean ± SEM; n=10 for WT and CaMKK2 KO. Statistical analyses were performed by repeated measures ANOVA. *p<0.01, WT vs KO.
